# Re-emerging threat of *Trypanosoma cruzi* vector transmission in El Salvador, update from 2018 to 2020

**DOI:** 10.1186/s40249-022-01008-5

**Published:** 2022-08-09

**Authors:** Marvin Stanley Rodríguez, Yuko Nitahara, Michelle Cornejo, Kevin Siliezar, Rafael Grande, Ana González, Kotaro Tasaki, Yu Nakagama, Yu Michimuko, Yoko Onizuka, Junko Nakajima-Shimada, José Eduardo Romero, José Ricardo Palacios, Carmen Elena Arias, William Mejía, Yasutoshi Kido, Ricardo Cardona Alvarenga

**Affiliations:** 1 Centro de Investigación y Desarrollo en Salud, Universidad de El Salvador, San Salvador, El Salvador; 2 Department of Virology and Parasitology, Graduate School of Medicine, Osaka Metropolitan University, 1-4-3 Asahi-machi, Abeno-ku, Osaka, 545-8585 Japan; 3 Research Center for Infectious Disease Sciences, Graduate School of Medicine, Osaka Metropolitan University, 1-4-3 Asahi-machi, Abeno-ku, Osaka, 545-8585 Japan; 4 Nagasaki University School of Medicine, Nagasaki, Japan; 5 Graduate School of Health Sciences, Gunma University, 3-39-22 Showamachi, Maebashi, Gunma 371-8514 Japan; 6 Ministerio de Salud, San Salvador, El Salvador; 7 Centro Nacional de Investigaciones Científicas de El Salvador, San Salvador, El Salvador; 8 Ministerio de Educación, Ciencia y Tecnología, San Salvador, El Salvador

**Keywords:** Chagas disease, Vector transmission, Triatomine, *Trypanosoma cruzi*, *Triatoma dimidiata*

## Abstract

**Background:**

Since the late twentieth century, Chagas disease gained global attention to suppress the vector burden as a main control strategy in endemic countries. In Central America, multi-national initiative successfully achieved significant reduction in the estimated disease prevalence as well as elimination of the region’s principal vector species at the time in 2012*.* While the last decade has witnessed significant changes in ecosystem—such as urbanization and replacement of the main vector species—that can possibly affect the vector’s habitation and residual transmission, the up-to-date vector burden in the region has not been evaluated thoroughly due to the cessation of active vector surveillance. The aim of this study was to update the risk of vector-borne *Trypanosoma cruzi* infection in El Salvador, the top Chagas disease-endemic country in Central America.

**Methods:**

A nationwide vector survey was conducted in the domestic environment of El Salvador from September 2018 to November 2020. The selection of the houses for inspection was based on expert purposeful sampling. Infection for *T. cruzi* was examined by microscopic observation of the insects’ feces, followed by a species confirmation using PCR. The data were analyzed using R software version 4.1.3. Proportion estimates with 95% confidence intervals were inferred using the Jeffrey’s method provided under the epiR package.

**Results:**

A total of 1529 *Triatoma dimidiata* was captured from 107 houses (infestation rate, 34.4%; 107/311) in all the fourteen departments of the country visited within the period; prevalence of *T. cruzi* infection was as high as 10% (153/1529). In the country, domestic *T. dimidiata* infestation was distributed ubiquitously, while *T. cruzi* infection rates varied across the departments. Five out of fourteen departments showed higher infection rates than the average, suggesting sporadic high-risk areas in the country.

**Conclusions:**

Our comprehensive study revealed substantial *T. cruzi* infection of *T. dimidiata* across the country, indicating potential active transmission of the disease. Therefore, strengthened surveillance for both vector and human infection is required to truly eliminate the risk of *T. cruzi* transmission in Central America.

**Supplementary Information:**

The online version contains supplementary material available at 10.1186/s40249-022-01008-5.

## Background

In Latin American countries, the infection of *Trypanosoma cruzi* predominantly occurs through the contact with contaminated feces of hematophagous triatomine bugs [1]. The vector burden reduction is considered the most effective strategy for Chagas disease control [2, 3]. Over the past decades, international control initiatives have put great efforts to suppress the domicile vector-borne transmission by combining indoor insecticides spraying, housing improvements and health education in many endemic countries [2].

In Central America, the Central American Initiative for Chagas Disease Control was launched in 1997 in coordination with Pan American Health Organization [4]. This multi-national initiative successfully achieved the certified elimination of *Rhodnius prolixus* in 2012, once considered as a major domestic vector species responsible for human infection [5, 6]. Consequently, the active transmission seemed interrupted in the region considering the reduction of estimated disease prevalence from 7% in the 1998 [4] to 1.3% in 2010 [1, 7]. Once recognized critically endemic, Chagas vector control in Central America is now considered under passive surveillance phase. The surveillance focuses on self-reported vector detection and spraying of residual infesting sites of triatomine bugs [2].

The transition of control program, however, may have resulted in neglecting the expansion of potential vector-borne *T. cruzi* transmission via emergence of a new vector species in the region: *Triatoma dimidiata*. This species was once found most prevalent in the sylvatic areas of Central America but now encountered widely in both rural and urban areas. This contrasts to *R. prolixus*, which was found exclusively in the domestic areas of rural residentials [6]. After the elimination of *R. prolixus* from Central America, the main contributor to vector-borne transmission has become *T. dimidiata.* In this study, we aimed to assess current infected vector burden by conducting a nation-wide survey in El Salvador. El Salvador is known to be one of the most endemic countries in the Central American region, with fluctuating seroprevalence around 2% among blood donors from 2001 to 2011 [8].

## Methods

### Nation-wide survey

Nation-wide active surveillance of the insects in residential areas of El Salvador was conducted between September 2018 and November 2020 by experienced field investigators of Centro de Investigación y Desarrollo en Salud (CENSALUD, San Salvador) and Ministry of Health of El Salvador. Detailed information of survey sites can be found in the Additional file 1. The selection of the houses for inspection was based on expert purposeful sampling of the field investigators. Briefly, area selection for the survey was based on following two components: (1) the materials of the houses were reported to be adobe, mud wall (“bajareque” in local language), or other natural materials and (2) area where infestation of triatomines was reported by residents through the Ministry of Health. The “man-hour” technique was used for insect collection; a total of one hour (or an hour divided by the number of investigators, if more than two) was spent for triatomine inspection [9].

### Vector identification and parasite detection

The insects’ species was determined morphologically by experienced entomologists at the CENSALUD laboratory. *T. cruzi* infection of the collected insects was determined by the conventional microscopic examination (under 400 ×) of insect feces mixed with normal saline solution. The routine surveillance of infected triatomine vector in the country through the Ministry of Health is completed by microscopic examination, mainly due to the shortage of instruments and resources to perform molecular diagnosis. Therefore, in this study, we adopted PCR diagnosis for molecular confirmation of *T. cruzi* species for the samples detected as positive under microscopic examination and a subset of microscopic negative samples to seek for the under-reported risk*.*

### DNA extraction and PCR protocol

DNA extraction assay was performed using DNEasy Blood and Tissue kit (QIAGEN, Germany), following the manufacturer’s procedure provided in the kit. Briefly on tissue preparation, approximately 25 μg of tissue from the back of the triatomine was cut with a leaf of new scalpel for each sample. The cut portion of the tissue was placed in a 1.5 ml tube with 200 μl of tissue lysis buffer (Buffer ATL, accompanied in the mentioned kit), followed by maceration with a homogenizer (Bio-Gen PRO200 Homogenizer, Pro Scientific, USA).

The PCR was conducted using 121 and 122 primers (121: 5′-AAA TAA TGT ACG GG(T/G) GAG ATG CAT GA-3′, 122: 5′-GGT TCG ATT GGG GTT GGT GTA ATA TA-3′) targeting specifically the kinetoplast minicircle DNA sequences [10, 11]. Each reaction was performed in a total of 25 μl reaction volume containing 0.5 μmol/L of each primer, 12.5 μl of GoTaq DNA polymerase (Promega, USA), 5 μl of DNA template, and additional distilled water to the final reaction volume. Briefly, the PCR condition was: 3 min at 94 °C for the denaturation, followed by 35 cycles of 30 s at 94 °C, 30 s at 55 °C, and 30 s at 72 °C with a final extension of 10 min at 72 °C. The amplified PCR product was detected at the size of 330 bp in the electrophoresis using 3% of agarose gel.

### Data analysis

The data were entered into Microsoft Excel spreadsheets (Microsoft Corp., USA, 2010) and analyzed using R software version 4.1.3 (The R Development Core Team, R Foundation for Statistical Computing, Austria). Proportion estimates with 95% confidence intervals were inferred in defined populations using the Jeffrey’s method provided under the epiR package [12]. This is one of the recommended methods for small-size datasets that provides reliable coverage and whose rationale has been described in detail elsewhere [13].

## Results

A total of 1529 triatomines were captured in 107 houses and house annexes of all fourteen departments of El Salvador between September 2018 and November 2020. A total of 311 houses were visited during the period, indicating 34.4% of infestation rate. The materials of 107 houses infested with *T. dimidiata* were: adobe (56.1%, 60/107), mud wall (16.8%, 18/107), brick (14%, 15/107), and others (13.1%, 14/107) (See Additional file 2 for more details).

Morphological examination revealed *T. dimidiata* among all the triatomines captured in the study. During the investigation period, 68, 1413, and 48 were captured each year respectively (See Additional file 3 for details of triatomines). 10.0% of *T. dimidiata* (153/1529) revealed parasite positivity under microscopic examination. All microscopic positive specimens and a subset of 130 *T. dimidiata*, randomly picked from 1376 microscopically negative *T. dimidiata,* underwent DNA extraction of the midgut content, and were used as template DNA in the PCR assay. As a result, 100% of microscopic positive specimens revealed molecular confirmation of *T. cruzi*. In the subset, 6.9% (9/130, 95% confidence interval: 3.7–12.6%,) identified positivity for *T. cruzi* by PCR. Considering these results, no less than 10.0% of *T. dimidiata* infested in domestic environment in El Salvador was *T. cruzi* positive by both microscope observation and PCR, indicating persistent vector-borne transmission risk in the region.

**Fig. 1:**
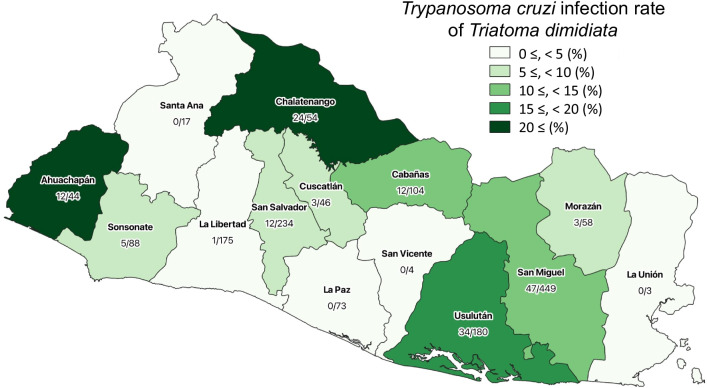
*Trypanosoma cruzi* infection rates of *Triatoma dimidiata* by department. Numbers below the name of each department indicate number of *T. cruzi*-positive *T. dimidiata* / total number of *T. dimidiata* captured in the department during the period of 2018 to 2020. Geographical data was obtained from https://www.diva-gis.org/datadown and depicted using QGIS software version 3.16.8-Hannover (http://www.qgis.org)

Geographical distribution of microscopic *T. cruzi-*positive *T. dimidiata* is shown in the country map (Fig. 1). Five out of fourteen departments showed higher infection rates than average of 10.0%, reflecting sporadic transmission risk in the country.

## Discussion

*T. dimidiata*, the native triatomine species to the Central American region, was once encountered mainly in sylvatic forests [6]. Since the elimination of *R. prolixus,* the former vector species, *T. dimidiata* has enlarged its habitation to domestic areas [5, 6]. As this study warrants, *T. dimidiata* is now observed ubiquitously, whether urban or rural, in the domestic environment of urban and rural areas of El Salvador. Other studies also show that *T. dimidiata,* having adapted to the environment change of urbanization in rural areas, may be responsible for the increase in human-vector contact [2, 14]. After all, the emergence of *T. dimidiata* as the principal vector has become a serious issue in the era of passive vector surveillance. However, the underlying vector burden with the emerging triatomine species, *T. dimidiata*, has not received enough attention. In fact, once there was plenty of reports on the vector prevalence through program-based surveys in the late 1900s, now few investigations focus to evaluate the current situation of vector-borne risk in the Central American region [1, 15]. From the recent available studies conducted partially in two villages in El Salvador, the infestation rate was less than 10% in 2011 [16]. Our active surveillance nationwide resulted in high infestation rate, possibly resulting from the mentioned purposeful sampling method. In this study, we evaluated the triatomine infection by microscopic observation, followed by molecular confirmation using PCR. Although we observed the submicroscopic *T. cruzi* infection in triatomine feces, a few percent of potential PCR positivity in microscopic negative samples might have less impact on the surveillance by the microscopic detection since physiological and pathological significance of submicroscopic infection in triatomines are yet to be defined.

Recent spread of immigrants from endemic countries changed the epidemiology of the disease to an emerging health issue in non-endemic countries as well [17, 18]. Many emigrants travel from Central American countries to non-endemic countries every year. Continued vectorial contamination risk in this region shall be recognized as potential health issues even in non-endemic countries [19].

## Conclusions

In this study, we analyzed the current potential transmission risk of *T. cruzi* via emerging vector of the region, *T. dimidiata.* We conclude that the substantial vector-borne transmission risk still exists in this region; no less than 10% of triatomine bugs are potentially transferable of *T. cruzi* to humans. Therefore, strong surveillance for both vector and human infection shall be required to eliminate the risk of *T. cruzi* transmission in Central America. Understanding the prevalence of the *T. cruzi* infected triatomine bugs in each area is crucial in order to evaluate area-specific risk of vector-borne transmission. With this information, cost optimization of the vector surveillance strategy is possible by targeting the areas with especially high-risk of vector-borne *T. cruzi* infection, which is a significant consideration in low- and middle-income countries limited of human, material, financial and administrative resources.

## Supplementary Information

**Additional file 1. **Details of survey area in this study.

**Additional file 2. **Wall materials of infested houses observed in this study.

**Additional file 3.** Details of triatomines captured in this study.

## Data Availability

All data generated or analyzed during this study are included in this published article and its supplementary information files.

## Abbreviations

CENSALUD: Centro de Investigación y Desarrollo en Salud
DNA: Deoxyribonucleic acid
PCR: Polymerase chain reaction
